# IgG4-related disease in patients with idiopathic orbital inflammation

**DOI:** 10.1186/s12886-021-02115-x

**Published:** 2021-10-08

**Authors:** Orapan Aryasit, Nanida Tiraset, Passorn Preechawai, Kanita Kayasut, Nuttha Sanghan, Wantanee Sittivarakul

**Affiliations:** 1 Department of Ophthalmology, Faculty of Medicine, Prince of Songkla University, Hat Yai, Songkhla, 90110 Thailand; 2 Anatomical Pathology Unit, Department of Pathology, Faculty of Medicine, Prince of Songkla University, Hat Yai, Songkhla, 90110 Thailand; 3 Department of Radiology, Faculty of Medicine, Prince of Songkla University, Hat Yai, Songkhla, 90110 Thailand

**Keywords:** IgG4-related disease, Idiopathic orbital inflammation, Corticosteroids, Prevalence, Infraorbital nerve enlargement

## Abstract

**Background:**

To identify the prevalence of positive IgG4 immunostaining in orbital tissue among patients previously diagnosed with nongranulomatous idiopathic orbital inflammation (IOI) and to compare the clinical characteristics of patients with and without IgG4-positive cells.

**Methods:**

A retrospective review of all patients with a histopathologic diagnosis of IOI was performed. Immunohistochemical staining was performed to identify IgG-positive cells and IgG4-positive cells. Multivariate analysis was performed using likelihood ratio-test logistic regression on the differences between IgG4-related disease (IgG4-RD) and non-IgG4-RD.

**Results:**

Of the 45 patients included, 21 patients (46.7%) had IgG4-positive cells, with 52.4% being male and a mean age of 55.9 ± 13.4 years. Bilateral ocular adnexal involvement (adjusted odds ratio [aOR] = 9.45; *P* = 0.016) and infraorbital nerve enlargement (aOR = 12.11; *P* = 0.008) were frequently found in IgG4-RD patients. Complete remission occurred in 23.8% of IgG4-RD patients and 41.7% of non-IgG4-RD patients. IgG4-RD patients had more frequent recurrent disease than non-IgG4-RD patients.

**Conclusions:**

Nearly 50% of IgG4-RD patients were previously diagnosed with biopsy-proven IOI. IgG4-RD was more frequent in patients with bilateral disease and infraorbital nerve enlargement, showing the importance of tissue biopsy in these patients. Immunohistochemistry studies of all histopathology slides showing nongranulomatous IOI are highly recommended to evaluate for IgG4-RD.

## Background

Idiopathic orbital inflammation (IOI), previously named orbital pseudotumor, is a benign, noninfective, tumefactive lesion of the orbit. Inflammatory clinical syndrome is characterized by features of a nonspecific or nonidentifiable local or systemic inflammatory condition [1, 2]. IOI is a diagnosis of exclusion and comprises cases with heterogeneous clinicopathology [3–5]. IOI is the third most common orbital disease, following Graves’ ophthalmopathy and lymphoproliferative diseases [6, 7]. IOI accounts for 5.2–11.0% of orbital disorders [8, 9].

The pathogenesis of IOI has remained inconclusive. Various lines of evidence point to an immune-mediated process as the main mechanism [10, 11]. In recent years, IgG4-related disease (IgG4-RD) has emerged as a common cause of orbital inflammation, suggesting that IOI might be a part of the disease spectrum of IgG4-RD [12, 13]. The exact prevalence of IgG4-RD is difficult to estimate since there have been few published studies, and these studies used different criteria for diagnosis, tended to enroll small groups of patients with heterogeneous pathology, and had uncertain generalizability [4].

The aim of this study was to determine the prevalence of positive immunostaining of IgG4 in patients who were previously diagnosed with biopsy-proven nongranulomatous IOI. We also compared the clinical features and treatment outcomes of patients with and without biopsy-proven IgG4-RD.

## Methods

### Study population

The electronic medical records of the patients initially diagnosed with biopsy-proven nongranulomatous IOI and treated at Songklanagarind Hospital from October 1, 2006, to September 30, 2016, were reviewed. This study was approved by the Research Ethics Committee and was performed in full compliance with the guidelines of the Declaration of Helsinki and the International Conference on Harmonization in Good Clinical Practice Guidelines (REC number 59–040–02-1). During the study period, tissue biopsies were not the first diagnostic step for all patients with IOI in our center. The inclusion criteria included all patients aged ≥18 years with biopsy-proven IOIs. Patients with a final diagnosis of other identifiable diseases, including Graves’ autoimmune disease, granulomatosis with polyangiitis (Wegener granulomatosis), lymphoma, and orbital cellulitis, were excluded from this study. We also excluded patients who had a follow-up time of less than 6 months and poor-quality histopathologic slides.

### Data collection

Data were reviewed, including age, sex, affected eye, visual acuity (VA), signs and symptoms, the location and extent of the disease, radiologic data, histopathologic features, and treatment outcome. Blood eosinophilia was defined as a percentage of eosinophils > 10 [14].

Serial 3-μm-thick sections were sliced from formalin-fixed, paraffin-embedded tissue. Immunohistochemistry staining was performed using anti-IgG (rabbit polyclonal, dilution 1:2000; CELL MARQUE, California, United States) and anti-IgG4 (mouse monoclonal, dilution 1:100; CELL MARQUE, California, United States) antibodies. All histopathologic specimens that revealed lymphoplasmacytic infiltrates were reviewed by an expert pathologist (KK), and additional immunohistochemical staining was performed to identify IgG4-RD according to the new diagnostic criteria for IgG4-related ophthalmic disease, reported in 2014 by Goto et al. [15] (Table 1).

**Table 1:** Diagnostic criteria for IgG4-related ophthalmic disease^15^

Diagnostic criteria for IgG4-related ophthalmic disease	
1. Imaging studies show enlargement of the lacrimal gland, trigeminal nerve, or extraocular muscle, as well as masses, enlargement, or hypertrophic lesions in various ophthalmic tissues.	
2. Histopathological examination shows marked lymphocyte and plasmacyte infiltration and sometimes fibrosis. A germinal center is frequently observed. IgG4 + plasmacytes are found and satisfy the following criteria: ratio of IgG4 + cells to IgG + cells of 40% or above, or > 50 IgG4 + cells per high-power field (× 400).	
3. Blood test shows elevated serum IgG4 (> 1.35 g/L).	
Definite: 1 + 2 + 3, Probable: 1 + 2, Possible: 1 + 3.	

For the tissue specimens of each patient, we selected three high-power fields (HPFs) with the highest concentration of IgG4-positive cells and IgG-positive cells, which were evaluated and counted. The average number of IgG4-positive cells per HPF and the average ratio of IgG4-positive to IgG-positive cells were then calculated. All orbital images were reported by the neuroradiologist (NS). Infraorbital nerve enlargement was evaluated using coronal sections of orbital computed tomography or magnetic resonance imaging scans. The criteria for the diagnosis of infraorbital nerve enlargement was abnormal findings including a loss of fat planes around the infraorbital nerve, focal or diffuse alteration of the caliber, irregular course, an expansion of the infraorbital canal or foramen, an enhancing infraorbital nerve, and asymmetrical size and signal intensity [16].

### Main outcome measures

The primary outcome measure was the prevalence of IgG4-RD in the case of previously diagnosed IOI. The secondary outcome measure was the difference between histopathologically confirmed IgG4-RD and non-IgG4-RD in terms of the clinical features and treatment outcomes. The treatment outcomes were classified into three categories: complete remission, partial response, and disease recurrence. Complete remission was defined as complete clinical remission, a lack of disease activity for 90 or more days with steroid or immunosuppressant discontinuation without relapse. Partial response was defined as persistent disease with relapse within 90 days after the discontinuation of steroid or immunosuppressant treatment. Disease recurrence was defined as repeated episodes of disease separated by periods of inactivity after 90 or more days without steroid treatment.

### Statistical analysis

Data were analyzed using Stata Statistical Software (STATA MP 14.1 StataCorp LP). Clinical characteristics between the IgG4-RD and non-IgG4-RD groups were compared using Student’s t test for continuous variables and Fisher’s exact test for categorical variables. In the analysis of histologic features, the chi-square test and Fisher’s exact test were used to compare categorical variables across groups. One-way analysis of variance (ANOVA) was used to compare means between groups. The risks for predicting IgG4-RD was determined by calculating the odds ratio (OR). Multivariate analysis was performed using likelihood ratio-test logistic regression on the differences between the IgG4-RD and non-IgG4-RD groups. Statistical significance was defined as *P* < 0.05.

## Results

### Study population

Sixty-five patients underwent tissue biopsy, and 20 patients were subsequently excluded (follow-up time of < 6 months [*n* = 13]; poor quality of histopathologic slides [*n* = 7]). Of the 45 patients who fulfilled the inclusion and exclusion criteria for this study, 21 patients (46.7%) had IgG4-positive cells, with 52.4% being male with a mean age of 55.9 ± 13.4 years, while the mean age at diagnosis of patients with IgG4-negative cells was 50.3 ± 17.1 years, and the patients were mainly female (79.2%). The mean logMAR of VA at diagnosis for IgG4-RD and non-IgG4-RD patients was 0.36 ± 0.33 and 0.39 ± 0.55, respectively. The most common presenting sign and symptom of both groups was a palpable mass or localized swelling in IgG4-RD and non-IgG4-RD patients (90.5 and 70.8%, respectively). Of the 21 IgG4-RD patients, 4 had lymph node enlargement. Between October 1, 2006, and March 25, 2014, serum IgG4 level testing was not available in our center. Only 5 out of 65 patients were tested to determine their serum IgG4 concentration, and all 5 patients had a concentration higher than 1.35 g/L. An imaging study was available for review in the picture archiving and communication system for 37 patients (82.2%). The most common imaging was computed tomography (70.3%), followed by magnetic resonance imaging (29.7%).

### Histopathologic features

The mean number of IgG-positive cells in IgG4-RD patients was 251.2 ± 172.4 cells, while the mean number of IgG-positive cells in non-IgG4-RD patients was 108.3 ± 119.6 cells (*P* = 0.002). The mean number of IgG4-positive cells in IgG4-RD and non-IgG4-RD patients was 138.1 ± 108.3 cells and 9.4 ± 13.2 cells, respectively (*P* < 0.001). The mean ratios of IgG4-positive cells to IgG-positive cells in patients with IgG4-RD and non-IgG4-RD were 56.3 ± 18.3 and 6.0 ± 8.2, respectively (*P* > 0.001). In the IgG4-RD group, we found that 95.2% of patients had > 50 IgG4+ cells/HPF, 81.0% of patients had an IgG4-positive cell/IgG-positive cell ratio ≥ 40, and 76.2% of patients fulfilled both criteria. Based on histopathologic criteria and limited evidence of serum IgG4 levels, only 2 patients were diagnosed with definite IgG4-RD, and 19 were diagnosed with probable IgG4-RD. Three patients who were histopathologically confirmed to have non-IgG4-RD with elevated serum IgG4 levels were diagnosed with possible IgG4-RD.

### Univariate analysis

The results of the univariate analysis of the variables associated with IgG4-RD diagnosis are illustrated in Table 2. IgG4-RD was found predominantly in males (*P* = 0.032). The frequency of patients with bilateral ocular adnexal involvement tended to be higher in IgG4-RD patients than in non-IgG4-RD patients (*P* = 0.010). Twelve of the 13 IgG4-RD patients with an enlargement of the infraorbital nerve also had canal enlargement. Blood eosinophilia was found to be more common in IgG4-RD patients (*P* = 0.015).

**Table 2:** Univariate analysis for IgG4-RD diagnosis

Variables	Biopsy-proven diagnosis		*P* Value	Odds ratio (95% CI)	*P* Value
	Non-IgG4-RD (*n* = 24 patients/30 eyes)	IgG4-RD (*n* = 21 patients/33 eyes)			
Age (years)					
Mean ± SD	50.29 ± 17.05	55.90 ± 13.36	0.230		
Median (min, max)	49.5 (18, 88)	57.0 (20, 77)			
Age (years)					
< 54	13 (54.2)	7 (33.3)	0.231	1	
≥ 54	11 (45.8)	14 (66.7)		2.36 (0.70, 7.04)	0.164
Gender					
Male	5 (20.8)	11 (52.4)	0.035	1	
Female	19 (79.2)	10 (47.6)		0.24 (0.06, 0.88)	0.032
Laterality					
Unilateral	18 (75.0)	9 (42.9)	0.037	1	
Bilateral	6 (25.0)	12 (57.1)		4.00 (1.39, 11.53)	0.010
Duration of symptom presentation (days)					
Mean ± SD	216.32 ± 253.35	579.84 ± 712.05	0.031		
Median (min, max)	120 (3, 1080)	365 (7, 2555)			
Duration of symptom presentation (days)					
< 240	14 (63.6)	6 (31.6)	0.062	1	
≥ 240	8 (36.4)	13 (68.4)		3.79 (1.03, 13.91)	0.045
Initial VA (logMAR)					
Mean ± SD	0.39 ± 0.55	0.36 ± 0.33	0.776		
Median (min, max)	0.2 (0, 2.3)	0.3 (0, 1.7)			
Initial VA					
Better than 20/50	22 (73.3)	21 (63.6)	0.208	1	
20/50 to 20/160	5 (16.7)	11 (33.3)		1.30 (0.68, 7.76)	0.178
20/200 or worse	3 (10.0)	1 (3.0)		0.35 (0.03, 3.63)	0.378
Pain					
No	19 (79.2)	16 (76.2)	1.000	1	
Yes	5 (20.8)	5 (23.8)		1.19 (0.29, 4.85)	0.811
Swelling/mass					
No	7 (29.2)	2 (9.5)	0.143	1	
Yes	17 (70.8)	19 (90.5)		3.91 (0.71, 21.46)	0.116
Diplopia					
No	21 (87.5)	19 (90.5)	1.000	1	
Yes	3 (12.5)	2 (9.5)		0.74 (0.11, 4.90)	0.752
Proptosis					
No	8 (33.3)	5 (23.8)	0.528	1	
Yes	16 (66.7)	15 (76.2)		1.60 (0.43, 5.96)	0.484
Red eye/chemosis					
No	19 (79.2)	12 (57.1)	0.196	1	
Yes	5 (20.8)	9 (42.9)		2.85 (0.77, 10.57)	0.117
Organ involvement					
Lacrimal gland	10 (41.7)	7 (33.3)	0.073	1	
Extraocular muscles	2 (8.3)	0 (0.0)		1 (omitted)	–
Anterior part	4 (16.7)	0 (0.0)		1 (omitted)	–
Apex	0 (0.0)	1 (4.8)		1 (omitted)	–
Diffuse pattern	8 (33.3)	13 (61.9)		2.32 (0.63, 8.58)	0.207
Structural involvement					
Single	15 (62.5)	7 (33.3)	0.075	1	
Multiple	9 (37.5)	14 (66.7)		3.33 (0.98, 11.37)	0.055
Enlargement of infraorbital nerve					
No	15 (83.3)	6 (31.6)	0.003	1	
Yes	3 (16.7)	13 (68.4)		10.83 (2.25, 52.20)	0.003
Eosinophils (%)					
Mean ± SD	5.31 ± 5.53	9.86 ± 5.54	0.009		
Median (min, max)	2.8 (0.0, 22.0)	10.1 (1.0, 20.0)			
Eosinophils (%)					
≤ 10	20 (83.3)	10 (47.6)	0.025	1	
> 10	4 (16.7)	11 (52.4)		5.50 (1.40, 21.71)	0.015
Final VA (logMAR)					
Mean ± SD	0.49 ± 0.75	0.25 ± 0.22	0.082		
Median (min, max)	0.15 (0.0, 3.0)	0.2 (0.0, 0.8)			
Final VA					
Better than 20/50	20 (66.7)	25 (75.8)	0.095	1	
20/50 to 20/160	6 (20.0)	8 (24.2)		1.07 (0.32, 3.58)	0.917
20/200 or worse	4 (13.3)	0 (0.0)		1 (omitted)	–
Treatment response					
Complete remission	10 (41.7)	5 (23.8)	0.262	1	
Partial response	6 (25.0)	4 (19.1)		1.33 (0.25, 7.01)	0.734
Intermittent episode	8 (33.3)	12 (57.1)		3.00 (0.74, 12.13)	0.123
Follow-up time (months)					
Mean ± SD	51.88 ± 39.32	58.79 ± 43.20	0.577		
Median (min, max)	44.37 (10.47, 163.40)	46.20 (7.53, 169.23)			

*IgG4-RD*: IgG4-related disease, *VA*: visual acuity, *CI*: confidence interval, *SD*: standard deviation

### Multivariate analysis

The results of multivariate analysis using likelihood ratio-test logistic regression showed that the significant clinical characteristics for IgG4-RD compared with non-IgG4-RD were bilateral ocular adnexal involvement (*P* = 0.016) and an enlargement of the infraorbital nerve (*P* = 0.008) (Table 3).

**Table 3:** Multivariate analysis for IgG4-RD diagnosis

Variables	Adjusted Odds Ratio (95% CI)	*P* Value (wald-test)	*P* Value (LR-test)
Age (years)			
< 54 year	1		
≥ 54 year	0.78 (0.11, 5.46)	0.799	0.798
Laterality			
Unilateral	1		
Bilateral	9.45 (1.10, 81.42)	0.041	0.016
Duration of symptom presentation (days)			
< 240	1		
≥ 240	1.99 (0.30, 13.14)	0.474	0.476
Swelling /mass			
No	1		
Yes	2.97 (0.23, 38.20)	0.384	0.380
Red eye/chemosis			
No	1		
Yes	3.04 (0.28, 32.44)	0.358	0.345
Enlargement of infraorbital nerve			
No	1		
Yes	12.11 (1.58, 92.51)	0.016	0.008
Structured involvement			
Single organ	1		
Multiple organs	2.71 (0.35, 20.69)	0.338	0.326

*IgG4-RD*: IgG4-related disease, *CI*: confidence interval, *LR*: likelihood ratio, *HPF*: High power field

### Treatment regimen

Of the 45 patients, 41 (91.1%) patients received oral prednisolone in various doses. Oral prednisolone was given with an initial dose of 1 mg/kg/day or 60 mg/day for 2 weeks, which was then tapered by 5–10 mg every 2–4 weeks, adjusting the duration according to the patient’s response. Seven patients (15.6%) received high doses of intravenous corticosteroids due to severe visual loss associated with optic nerve involvement or severe bilateral inflammation, with a mean logMAR VA of 0.50 ± 0.55, whereas the mean logMAR VA of patients who received oral prednisolone was 0.38 ± 0.51 (*P* = 0.259). This study revealed that only 1 IgG4-RD patient received orbital radiation and showed a good response, and 3 non-IgG4-RD patients received orbital radiation and showed a good response. Methotrexate was used to treat 5 IgG4-RD patients and 2 non-IgG4-RD patients who had elevated serum IgG4 levels and were diagnosed with possible IgG4-RD. Observation was reported for 1 patient with IgG4-RD and 2 patients with non-IgG4-RD due to the refusal of treatment.

### Treatment outcome

The mean follow-up time in this study was 58.8 ± 44.2 months (range, 7.5–169.2 months) in the IgG4-RD group and 51.9 ± 39.3 months (range, 10.5–163.4 months) in the non-IgG4-RD group. Among the 45 patients, complete resolution was reported in 15 patients (33.3%).

In IgG4-RD patients, on the basis of treatment response, only 23.8% of patients had a complete resolution of their symptoms and signs with one round of treatment, 57.1% of patients had a recurrence of disease. Ten (41.7%) out of 24 non-IgG4-RD patients had a complete response. The mean age of the IgG4 patients with complete remission was 65.20 ± 8.35 years, which was slightly higher than that of the noncomplete remission group (mean, 52.80 ± 16.03 years).

Overall, patients in the incomplete remission group received higher doses **(**1 mg/kg/day**)** of oral prednisolone than those in the complete remission group **(**85**.**1**%** versus 65**.**6**%**; *P* **=** 0**.**714**).** Seven patients **(**5 IgG4-RD patients and 2 non-IgG4-RD patients**)** were treated with methotrexate **(**6 with a good response and 1 being lost to follow-up**).** The treatment outcome in the patients with IgG4-RD tended to involve more disease recurrence or more intermittent episodes than in the non-IgG4-RD patients.

## Discussion

This study demonstrated that IgG4-RD accounted for nearly half of the total cases previously classified as biopsy-proven nongranulomatous IOI. We observed that bilateral ocular adnexal disease and an enlargement of the infraorbital nerve were more frequent in IgG4-RD than in non-IgG4-RD patients.

In agreement with previous studies, IgG4-RD showed similarities with several of the clinical characteristics of IOI, which, in general, tends to affect men more than women [3, 14, 17]. Our study revealed that non-IgG4-RD patients were mostly female, which is similar to the findings in published studies [1, 18]. Bilateral ocular adnexal involvement tended to be significantly more common in IgG4-RD than in non-IgG4-RD patients, with an aOR of 9.45 (*P* = 0.016). This correlates with recent evidence suggesting that bilateral disease should be considered for IgG4-RD [19–21]. Additionally, IgG4-RD patients who had bilateral disease potentially had extraophthalmic involvement [22]. We also found that diffuse lesions and multiple structural involvements of the orbit were more frequent among IgG4-RD patients. None of the IgG4-RD patients presented with pure myositis or pure anterior orbital involvement. A study from the United States reported that in 21 patients with IgG4-RD, the lacrimal gland was the most commonly involved site (13/21, 61.9%) [23].

Infraorbital nerve and canal enlargement was strong evidence for a diagnosis of orbital reactive lymphoid hyperplasia and IgG4-RD with concurrent orbital myositis, focal orbital disease, or paranasal sinus disease [24–26]. Additionally, infraorbital nerve enlargement in IgG4 patients was associated with higher serum IgG4 [12]. This study also reported a strong association between IgG4-RD and infraorbital nerve enlargement, which was one of the significant clinical indicators of IgG4-RD. However, the main orbital imaging in our study was computed tomography. Therefore, the definition of infraorbital nerve enlargement in our study was different from that of the previous study that defined infraorbital nerve enlargement as this nerve being larger than the optic nerve in the coronal section of orbital magnetic resonance imaging scans [26].

Previous published studies on pathologic characteristics have advocated a strong relationship between IOI and IgG4-RD [4, 14, 19]. We found a high frequency of biopsy-proven IgG4-RD among patients with previously diagnosed IOI. The frequency of IgG4-RD in patients with IOI varies from 5.4 to 45.8% [4, 14, 21]. Such discrepancies between prior studies in the rates of IgG4-RD are possibly due to differences in diagnostic criteria retained for tissue IgG4 plasma cell infiltrates (i.e., either > 10 IgG4+ cells/HPF [27], > 30 IgG4+ cells/HPF [28], or > 50–100 IgG4+ cells/HPF [29]). Additionally, an elevated ratio of IgG4+ cells to IgG+ cells, the most sensitive and specific histopathologic parameter, was considered when the difference in these cell counts was more than 40% [30]. A limitation of the use of the average number of IgG4+ plasma cells counted was also recognized because of the patchy distribution of IgG4+ cells, the variability in the size of an HPF across microscopes, and the relatively low specificity of IgG4+ cell counts [31]. However, there were no differences between the use of one criterion and the use of both criteria for diagnosis. We suggest following the diagnostic criteria reported in 2014 [15]. Our study also revealed significantly more IgG+ cells/HPF in patients with IgG4-RD; therefore, pathologists should be concerned with additional evidence of IgG4-RD. Histopathologic features of ocular adnexal IgG4-RD reveal the similarity of the other sites, except in the case of storiform fibrosis and obliterative phlebitis, which were less common in several prior studies [4, 32]. Therefore, we focused on the IgG4+ cell to IgG+ cell ratio and the number of IgG4+ cells.

We reported that IgG4-RD accounted for a high prevalence of cases originally diagnosed as IOI. The reasons for the higher frequency of probable IgG4-RD may be due to the following facts. First, the study was performed in a tertiary-care hospital; thus, more severe cases were included. Second, some patients underwent multiple incisional biopsies due to partial response to treatment or a recurrence of inflammation. We used the most severe tissue specimen as the representative specimen. Third, we tended to perform incisional biopsy in patients with atypical presentations or those who failed to respond to the initial therapy rather than in patients with classic IOI cases, which might have led to a selection bias.

For the treatment of previously diagnosed IOI in the current study, most patients were treated with systemic corticosteroids, primarily at various doses. The majority of patients initially received oral prednisolone 1 mg/kg/day with a low chance of complete resolution. High doses of intravenous corticosteroids were used for patients with severe visual loss associated with optic nerve involvement and severe orbital inflammation to hasten inflammation reduction. However, there was no consensus on the treatment protocol, and the choice of therapy needs to be individualized by ophthalmologists and physicians with experience using these drugs in ocular inflammatory disease [20]. This high rate of initial response to prednisone is a common feature of IgG4-RD. However, recurrence, partial response, and severe prolonged inflammation with this disease were frequent (roughly three-quarters of patients). Our study demonstrated that IgG4-RD tended to be recurrent or had more intermittent episodes than non-IgG4-RD, although the difference was not statistically significant (57.1% versus 33.3%).

Five tissue-proven IgG4-RD and 2 possible IgG4-RD patients were treated with methotrexate. Therefore, ophthalmologists preferred prescribing steroid-sparing agents in patients with intractable orbital inflammation, including IgG4-RD, which was more prevalent in elderly patients with frequent comorbidity and polypharmacy [33]. Methotrexate was safe and cost-effective in patients with IgG4-RD [34]. Hence, it is necessary to investigate the diagnosis of IgG4-RD, which may require more aggressive treatments or the maintenance of low-dose systemic steroid treatment to prevent disease recurrence [35].

The strength of the present study was its enrollment of nongranulomatous IOI patients and focus on the significant clinical differences between IgG4-RD and non-IgG4-RD, including treatment outcomes with long-term follow-up. We could determine the predictors for the diagnosis of IgG4-RD in terms of demographic data, imaging studies, and laboratory features. To the best of our knowledge, this study is the first to determine clinical differences between IgG4-RD and non-IgG4-RD using multivariate analysis. Clinical indicators of IgG4-RD were bilateral disease and an enlargement of the infraorbital nerve. The findings from our report could provide more information on IgG4-RD to clinicians in a developing country with a limited number of oculoplastic surgeons. Therefore, the clinical features could help in the formation of the diagnosis, especially in patients with suspected IgG4-RD in whom a surgeon cannot perform a tissue biopsy. Reportedly, lymphoid hyperplasia of IgG4-RD can provide a substrate for the emergence of lymphoma [36, 37]; thus, long-term observation is also warranted.

The data of this study were reviewed between 2006 and 2016. Nearly 50% of all biopsy-proven cases were previously diagnosed as IOI. Interestingly, IgG4-RD has been underestimated until now. Furthermore, the identification of IgG4-RD using serum IgG4 levels and immunohistochemistry staining in patients with suspected IgG4-RD is beneficial for more systemic investigation, optimal treatments, and adequate follow-up time. Prospective randomized controlled trials are required to determine the pattern of tissue involvement and histopathology in IgG4-RD, in addition to the development of effective treatment.

There were some limitations in our study. First, given the retrospective nature of this study, information bias arising from incomplete or missing data may be more pronounced than in a prospective study. Second, there was a lack of consistent laboratory testing for serum IgG4 levels when the patients underwent tissue biopsy, for which serum IgG4 concentration is one of the IgG4-RD diagnostic criteria. Therefore, there were few patients with definitive diagnoses of IgG4-RD in our study. If clinical features, orbital imaging, and pathologic findings met the diagnostic criteria, a probable diagnosis of IgG4-RD could be made. Last, we found that lymph node involvement was low because the lymph node status was assessed by manual palpation, which may have led to an underestimation of the rate of lymph node involvement. Additionally, we could not assess the association with extraorbital manifestations of IgG4-RD due to the low awareness of this disease in recent decades.

In summary, the results of our study revealed that IgG4-RD accounted for nearly half of cases initially diagnosed as biopsy-proven IOI. The main clinical features were strong recognition of IgG4-RD, including bilateral disease and an enlargement of the infraorbital nerve. Patients presenting with orbital inflammatory lesions should have biopsies obtained whenever possible and should be evaluated for IgG4-RD.

## Data Availability

The datasets used and/or analyzed during the current study are available from the corresponding author upon reasonable request.

## Abbreviations

IOI: idiopathic orbital inflammation IgG4-RD IgG4-related disease aOR adjusted odds ratio VA visual acuity HPFs high-power fields
